# Does Knowledge of the Partner's Need Affect Food Sharing in Tufted Capuchin Monkeys?

**DOI:** 10.1002/ajp.70083

**Published:** 2025-10-24

**Authors:** Gabriele Schino, Guendalina Francesconi, Elsa Addessi

**Affiliations:** ^1^ Istituto di Scienze e Tecnologie della Cognizione Consiglio Nazionale delle Ricerche Rome Italy; ^2^ Dipartimento di Biologia Ambientale Sapienza Università di Roma Rome Italy

**Keywords:** empathy, food sharing, replication, *Sapajus*

## Abstract

The ability to understand the internal (psychological or physiological) states of others can be adaptive in both cooperative and competitive settings. In this study, we tested whether tufted capuchin monkeys (*Sapajus* spp.) altered their willingness to share part of their food with a partner in relation to having recently seen it eating. We first gave partners food (banana, carrot, or nothing) that could not be shared with the subjects, and then gave subjects food (apple) that they could share with the partners. During the first phase of the tests, the subjects seemed aware that their partner was eating, but did not show any evident emotional response. Neither having seen the partner eat nor the quality of the food eaten by the partner had any effect on the subject's willingness to subsequently share their food during the second phase of the tests. Our results do not confirm those of a previous similar study, and suggest the effect of having seen the partner eat on subsequent food sharing is weak and/or variable in tufted capuchin monkeys. The ability of animals to understand the internal states of other individuals remains contentious.

## Summary

- Capuchins were given the possibility to share food after having or not having seen a partner eat.
- Capuchins were aware their partner was eating, and showed no sign of distress.
- Having seen their partner eat did not affect subsequent food sharing.
- Our results do not replicate a previous similar study.

## Graphical abstract

Capuchin monkeys were given the possibility to share food after having or not having seen a partner eat. Neither having seen the partner eat nor the quality of the food eaten by the partner had any effect on the monkeys' willingness to share part of their food.

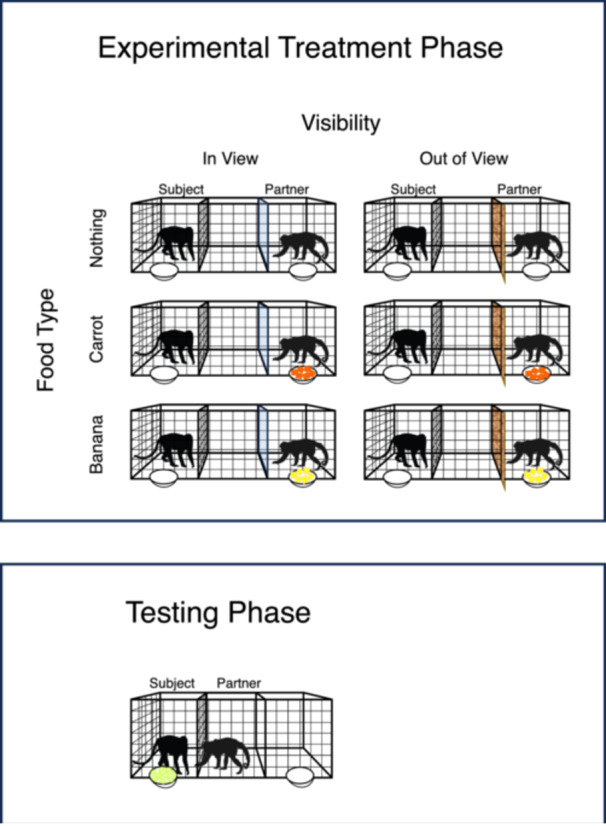

## 1 Introduction

Understanding that other individuals have internal states (physiological or psychological) distinct from one's own and that these internal states govern the actions of other individuals can have an obvious adaptive value both in cooperative and in competitive interactions (here we consider as cooperative any behavior that provides a benefit to another individual; West et al. 2007). The extent to which animals do have some knowledge of the internal states of others has been the focus of a large research effort, but our understanding of what exactly is known and the taxonomic distribution of this knowledge is still fragmentary (for reviews, see Adriaense et al. 2020; De Waal and Preston 2017; Legg et al. 2015). Understanding the physiological states, desires, and intentions of others may be the first step towards the evolution (and development) of more complex capacities such as perspective taking, attribution of (false) beliefs, and a full‐fledged theory of mind. Therefore, while the latter may be restricted to humans and perhaps to a few cognitively advanced nonhuman species (Call and Tomasello 2008), the simpler understanding of internal states may be expected to be found in a wider array of moderately large‐brained animals.

In a cooperative context, knowledge of the internal state of others may be inferred based on variations in the propensity to help in relation to the partner's need. A handful of studies investigated whether animals altered their willingness to provide food for a conspecific in relation to their knowledge about its presumed state of hunger/satiety. Ostojić et al. (2013) showed that male Eurasian jays (*Garrulus glandarius*) decreased provisioning of a specific food to their mate after having seen her eat that specific food. They ruled out alternative explanations, such as behavior reading by using visible/blind pretests (in which the female ate while being visible or not visible to the male) and concluded Eurasian jays demonstrate state attribution and provide evidence of self/other differentiation.

Other studies yielded less convincing evidence. Massen et al. (2020) reported that azure‐winged magpies (*Cyanopica cyanus*) shared a preferred food with conspecifics in relation to the availability of the same preferred food to the recipients. However, it is possible that the recipients were simply more interested in receiving food when they did not have direct access to it, as no attempt was made to control for this factor. Shaw et al. (2017) showed that male New Zealand robins (*Petroica longipes*) preferentially fed their mate a type of food that she had not previously been fed, but this effect seemed to depend on the female's behavior rather than on state attribution, since it did not vary in relation to the male having or not having seen the female eat.

Taborsky and colleagues conducted detailed analyses of the factors that facilitate food provisioning to a hungry partner in rats (*Rattus norvegicus*). They showed that rats increased their food provisioning in response to specific chemical and acoustic signals and to failed attempts to reach the food by the hungry partner (Schweinfurth and Taborsky 2018; Schneeberger et al. 2020; Paulsson and Taborsky 2021, 2022). Rats seem thus to adapt their sharing in relation to the need of the partner, but to do so on the basis of cues directly provided by the partner.

Surprisingly, little effort has been made to study similar phenomena in primates. Brügger et al. (2018) observed that cooperatively breeding marmoset monkeys (*Callithrix jaccus*) shared more food with immatures when no other group member was present, and speculated that in the absence of other group companions, immatures could be perceived as being in greater need of being helped. Mikeliban et al. (2021) observed that orangutan (*Pongo abelii*) mothers were more likely to share with their immature offspring food that was difficult to process, although this was possibly driven by increased solicitations by the offspring. Sehner et al. (2025) found that marmosets were also more likely to share with immatures food that was difficult to obtain than easily accessible food, and suggested cooperative breeding may be associated with an increased sensitivity to immature skill deficits. Finally, Hattori et al. (2012) tested whether tufted capuchin monkeys (*Sapajus* spp., formerly known as *Cebus apella*) altered their willingness to share food with a partner as a consequence of having seen it eat immediately before the test. They found that capuchin monkeys decreased food sharing when they had seen the partner eat shortly before the test, and proposed three possible interpretations for this finding. First, they reasoned, it is possible that the decrease in food sharing was, in fact, due to a decreased interest in obtaining food by the partner, which had already been partially satiated by the previous food consumption. Second, it is possible that seeing the partner eat induced negative emotions and an agonistic motivation in the subject, and that this negative attitude towards the partner lasted through the subsequent test and caused the decreased food sharing. Third, it is possible that the subject had some understanding of the partner's state of need (or absence of need) and that seeing the partner eat (or not eat) directly affected this knowledge and the willingness to empathically act (or not act) to reduce the partner's state of need (i.e., the subject's willingness to share food). Hattori et al. (2012) tested the first of these hypotheses by preventing the subject from seeing the partner eat (similarly to Ostojić et al. 2013). When the subject was unable to see the partner eat before the test, food sharing was unaffected by the partner's previous consumption of food, showing that changes in the partner's interest in obtaining food during the test did not explain the observed results. Hattori et al. (2012) also preliminarily discarded the second hypothesis on the basis of the observation that threatening behavior was rare and did not change across experimental conditions. They concluded that capuchin monkeys seemed to have some understanding of the state of need of the partner and altered their tolerance to food sharing in relation to this understanding.

A better test of the hypothesis that seeing the partner eat induced negative emotions and a hostile attitude in the subject could be obtained by varying the quality of the food eaten by the partner. It is possible to hypothesize that seeing the partner eat a low‐quality, less preferred food should induce a lower negative emotional response than seeing the partner eat a high‐quality, highly preferred food. The aim of our study was therefore twofold. First, we aimed at partially replicating the study of Hattori et al. (2012), thus testing, in an independent sample, whether seeing the partner eat alters the willingness of capuchin monkeys to share their food. The replication of scientific findings is a cornerstone of scientific research, and the current concern about the lack of reproducibility of much behavioral research makes it even more compelling (Pashler and Wagenmakers 2012; Open Science Collaboration 2015). Second, we aimed at testing the emotional/motivational hypothesis of Hattori et al. (2012) by letting our subjects see their partner eat food of variable quality, under the assumption that seeing a partner eat a preferred food should induce a stronger emotional response than seeing it eat a less preferred food.

Capuchin monkeys are particularly well‐suited to test the understanding of a partner's need in a cooperative context. They have a relatively large brain and cognitive capacities that often surpass those of other monkeys (Fragaszy et al. 2014). They are also fairly tolerant and readily share food, in particular in the experimental context we adopted (de Waal 1997; Hattori et al. 2012; Sabbatini et al. 2012; Schino, Ferrario, et al. 2021). It should also be noted that previous research in this same experimental context has shown that most food transfers are passive (the food possessor simply lets some food drop on the floor of the cage within reach of the partner), but that food possessors do retain some control on the amount of food received by the partner. This is shown by their ability to reciprocate the amount of food received and by their increased reluctance to share more preferred food (de Waal 2000; Sabbatini et al. 2012; Schino, Ferrario, et al. 2021).

Summarizing, we tested the following hypotheses and associated predictions.

Hypothesis 1 If capuchin monkeys alter their willingness to share part of their food in relation to knowledge about the state of hunger or satiation of their partner, then we expected: (1a) that the effect of the food eaten by the partner before the test on food transfers from the subject to the partner during the test should be moderated by the possibility for the subject to see the partner eat, regardless of the kind of food eaten by the partner; (1b) that, considering only the test sessions in which the subject had had the possibility to see the partner eating or not before the test, food transfers from the subject to the partner during the test should be less frequent when the partner had eaten (again, regardless of the kind of food eaten) than when it had not eaten any food.

Hypothesis 2 Should Hypothesis 1 be confirmed, if the observed effects are due to the subject experiencing negative emotions and hostility as a consequence of seeing the partner eat before the test, then we expected: (2) that the observed effects should be greater when the partner had eaten a preferred food than when it had eaten a less preferred food.

## 2 Methods

### 2.1 Subjects and Housing

Fifteen tufted capuchin monkeys living in the Primate Center of the Istituto di Scienze e Tecnologie della Cognizione, C.N.R., in Rome, Italy, participated in this study (Table 1). They lived in five social groups formed by 2–8 monkeys housed in separate outdoor enclosures connected to indoor rooms. Both the indoor rooms and the outdoor enclosures (measuring 40–130 m^2^, depending on group size) were equipped with perches, ropes, and tree trunks. The monkeys were fed vegetables, fresh fruits, and monkey chow in the afternoon (i.e., after testing), while water was always available. Foods used during the experiment (see below) were part of the normal diet of the monkeys.

**Table 1:** Subjects of the study.

Subject		Partner		Relatedness	Group membership
Name	Sex	Name	Sex		
Cognac	M	Gal*	M	Unrelated	1a
Totò*	M	Paprika	F	Unrelated	1b
Paprika	F	Totò*	M	Unrelated	1b
Robot*	M	Saroma	F	Unrelated	2
Sandokan*	M	Roberta	F	Unrelated	3
Roberta	F	Sandokan*	M	Unrelated	3
RobinHood*	M	Penelope	F	Unrelated	4
Penelope*	F	Peonia	F	Mother–daughter	4
Peonia	F	Penelope*	F	Daughter–mother	4
Quincy*	F	Pacajà	F	Daughter–mother	4
Rucola*	F	Robiola	F	Sisters	4
Robiola	F	Rucola*	F	Sisters	4

*Note:* An asterisk indicates the higher‐ranking member of each pair.

The monkeys were tested in pairs (a subject and a partner) and in most cases played both roles in separate tests (Table 1). The pairs were formed by “compatible” monkeys, that is, monkeys that would willingly enter together the indoor rooms. While this introduces a problem of self‐selection of subjects (i.e., tested pairs were not a random sample of all possible pairs), it was inevitable for both logistic and ethical reasons.

### 2.2 Experimental Procedure

The monkeys were tested in a set of three equally sized experimental cages connected to the indoor rooms. The experimental cages, each measuring 60 × 75 × 75 (height) cm, could be separated by wire mesh or solid (opaque or transparent) partitions. The experimental cages closest to and farthest from the indoor room were equipped with a cup hung to the bars of the cage's front side.

**Figure 1:**
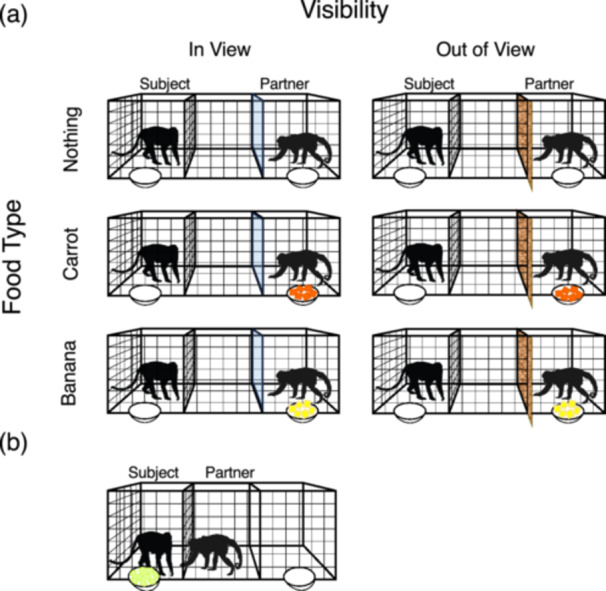
Phases and experimental conditions of the tests. (a) Experimental Treatment Phase: schematic representation of the six experimental conditions. The partner (in the rightmost of the three experimental cages) is given different types of food (nothing, carrot or banana) while in view or out of view of the subject; food sharing is prevented by the solid partition (transparent or opaque) separating the monkeys; (b) Testing Phase: the subject (in the leftmost of the three experimental cages) is given food (apple); food sharing is possible thorough the wire mesh separating the monkeys.

Figure 1 shows the experimental setting and summarizes the experimental conditions and experimental phases. Each test session consisted of two phases, an Experimental Treatment Phase and a Testing Phase. The Experimental Treatment Phase was meant to manipulate the feeding behavior of the partner (if and what the partner ate) and the subject's knowledge of such behavior. The Testing Phase aimed at measuring the effects of the experimental treatments on the subject's subsequent willingness to share part of its food.

During both phases, the subject was locked in the experimental cage farthest from the indoor room and separated from the central experimental cage by a wire mesh partition, while the partner could move freely between the indoor room and its experimental cages. During the Experimental Treatment Phase, the experimental cage closest to the indoor room was separated from the central cage by a solid partition that could be either opaque or transparent (according to condition, see below). The partner could be given either no food or 50 g of peeled and diced carrot or banana in the cup hung to the bars of the experimental cage closest to the indoor room. Given that the subject and the partner were separated by a solid partition, such food could not be shared. The Experimental Treatment Phase lasted 3 min, after which any remaining food was removed and weighed. Before the beginning of the Testing Phase, the solid partition was removed, so that the partner could access the central cage next to the subject, and the subject was given 150 g of peeled and diced apple in the cup hung to the bars of its experimental cage. The Testing Phase lasted 7 min, after which any food remaining in the cup was weighed. All tests were video‐recorded.

We manipulated two different experimental variables: the type of food given to the partner (Food Type: Nothing, Carrot, or Banana) and whether the partner was visible to the subject or not (Visibility: In View or Out of View), for a total of six different experimental conditions (Figure 1). Each subject was tested four times in each experimental condition, always with the same partner, for a total of 24 test sessions. The only exception was a single subject who refused to continue the experiment after 14 test sessions.

The order of presentation of the different experimental conditions was counterbalanced across dyads. Dyads were tested once a day, between 10:00 and 14:00. For monkeys that were tested in both roles (as subjects and as partners), at least a week elapsed between the end of a series of test sessions in one role and the beginning of the series of test sessions in the different role.

### 2.3 Data Collection and Analysis

Using the software BORIS (Friard and Gamba 2016), we scored from videos the behaviors described in Supporting Information S1: Table S1. We counted as food transfers all instances of collecting food in view, collecting food out of view, relaxed claim, food giving, and accidental food giving. A single observer scored all the videos. A second observer scored again 15% of the videos to assess interobserver reliability. Intraclass correlation coefficients ranged between 0.94 and 0.98.

We were interested in testing whether seeing the partner eat during the Experimental Treatment Phase affected the willingness of subjects to allow them to access their food during the subsequent Testing Phase, and whether this effect was dependent on the quality of the food being eaten by the partner (see the Supporting Information: S1 for details about food preferences). Food transfers from the subject to the partner, however, can also depend on the interest (hunger) of the partner, which can obviously be affected by food eaten during the Experimental Treatment Phase. This is why, following Hattori et al. (2012), we included a control condition in which the subject could not see the partner eating. The critical test of our hypothesis is thus a test of the interaction between the type of food (if any) eaten by the partner and the possibility for the subject to see the partner eat or not eat. It is also interesting, however, to simply test whether having seen the partner eat (or not eat) affects the subsequent food transfers.

Based on these considerations, we first ran analyses in which we tested whether the effects of food eaten by the partner during the Experimental Treatment Phase (Food Type) on the various dependent variables we examined were moderated by the visibility of the partner (Visibility). Then, we ran specific contrasts (Mitchell 2012) comparing the behavior observed when the partner had received no food while being visible to the subject (Food Type: Nothing; Visibility: In View) with that observed when the partner had eaten carrot or banana, again while being visible to the subject (Food Type: Carrot; Visibility: In View, or Food Type: Banana; Visibility: In View). Note that in the presence of an interaction (and if data had not been centered), main effects are calculated at the base level of the interacting variables. This is why in the tables reporting our results, *p*‐values of the main effect of Food Type are identical to those obtained in the specific contrasts (that were calculated at the base level of Visibility, i.e., when the partner was visible). We opted not to center the independent variables because the main effect of Food Type would in any case be meaningless, as it would represent the “average” effect of food received by the partner calculated including both test sessions in which the subject had had the possibility to see the partner eat and test sessions in which the subject was unaware that the partner had eaten.

Most of our analyses were within‐subject (fixed effect) conditional Poisson regressions with bootstrap (10,000 replications) standard errors in which each test session provided one data point. Within‐subject regressions allow the use of multiple data points per subject while controlling for between‐subject variation and avoiding pseudoreplication (Allison 2009). The dependent variable was the number of events (count) of the behavior being analyzed (e.g., food transfers). The independent variables were the type of food given to the partner during the Experimental Treatment Phase (Food Type: Nothing, Carrot, or Banana), the visibility of the partner during the Experimental Treatment Phase (Visibility: In View or Out of View), and their interaction. Exposure (offset) variables were entered as needed and specified in Supporting Information S1: Tables S2–S14.

We conducted a single linear regression (specifically, a within‐subject linear regression with bootstrap standard errors) to analyze the amount of food eaten by the partner during the Experimental Treatment Phase. The independent variables were the same as those used in the analyses described above.

We also conducted an additional within‐subject conditional Poisson regression to evaluate whether the partners' failed attempts to collect food were interpreted (and responded to) by the subjects as signals of need (Schweinfurth and Taborsky 2018; Paulsson and Taborsky 2021). In this analysis, the count of food transfers was the dependent variable, the count of failed attempts to collect food in view of the subject was the independent variable of interest and the type of food received by the partner during the Experimental Treatment Phase and the count of failed attempts to collect food out of view from the subject were further (control) independent variables.

To approximate what had been done by Hattori et al. (2012), we repeated our analysis on the effect of the experimental treatment on food transfers (described above), including only subsets of data. Specifically, we ran analyses that included: (1) only the first test session for each experimental condition; (2) only female‐female dyads; (3) only food transfers that occurred in view of the subject; (4) only test sessions in which the partner spent at least 70% of time in the experimental cages; (5) only test sessions in which the partner spent at least 90% of time in the experimental cages.

All statistical analyses were run using Stata 17.0 (StataCorp 2021).

## 3 Results

Throughout this section, we present only the results relevant to the test of our hypotheses (see above). Complete regression tables are included in Supporting Information: S1.

In two out of a total of 278 test sessions, the partner did not eat any of the food received from the experimenter (in both cases, carrot). Excluding those two test sessions from analysis did not change the results.

### 3.1 Behavior During the Experimental Treatment Phase

We first tested whether, during the Experimental Treatment Phase, the feeding behavior of partners differed when they were given carrots or bananas (this first analysis obviously excluded the test sessions in which no food was given to the partner). The difference in the amount of carrot or banana eaten was not affected by whether partners were visible to the subjects (interaction effect between Food Type and Visibility: Coeff. = 1.11, *z* = 0.51, *N* = 184, *p* = 0.607; Supporting Information S1: Table S2). Considering only the test sessions in which the partner was visible to the subject, partners ate more bananas than carrots (*χ* ^2^ = 38.69, *df* = 1, *p* < 0.001). Similarly, the difference in time spent eating carrots or a bananas was not affected by whether or not partners were visible to the subjects (interaction effect between Visibility and Food Type: Coeff. = −0.01, *z* = −0.18, *N* = 184, *p* = 0.859; Supporting Information S1: Table S3). However, considering only the test sessions in which the partner was visible to the subject, time spent eating bananas and carrots did not differ (*χ* ^2^ = 0.04, *df* = 1, *p* = 0.838). Thus, partners ate more bananas than carrots and ate them faster, confirming that bananas were the preferred food.

**Figure 2:**
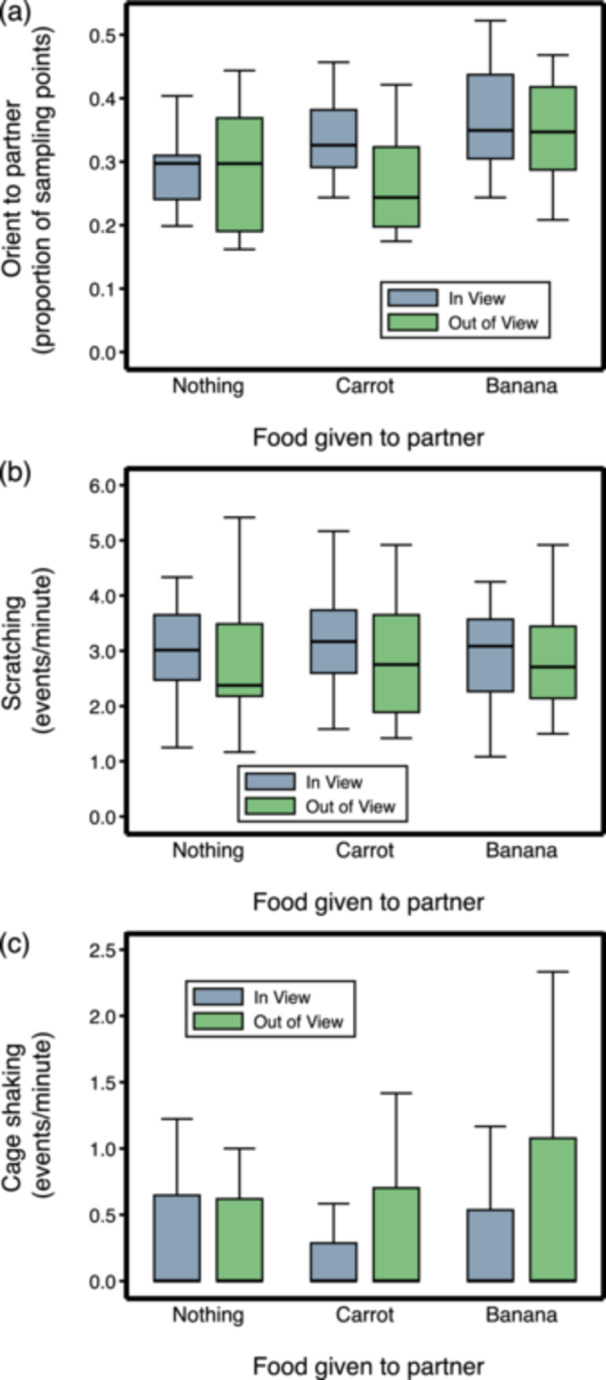
Behavior of subjects during the Experimental Treatment Phase in relation to food eaten by their partner (box plots showing medians, interquartile ranges, and adjacent values). (a) Time spent oriented towards the partner; (b) Scratching; (c) Cage shaking (note that medians for cage shaking were always zero).

Then we tested whether subjects realized that their partner had been given food (i.e., we tested for task understanding). Visibility moderated the orientation of the subjects towards their partner when the latter ate carrots (interaction effect between Visibility and Nothing vs. Carrot: Coeff. = −0,25, *z* = −2.02, *N* = 278, *p* = 0.044), but not when it ate bananas (interaction effect between Visibility and Nothing vs. Banana: Coeff. = −0,07, *z* = −0.82, *N* = 278, *p* = 0.414; Supporting Information S1: Table S4 and Figure 2a). Considering only the test sessions in which the partner was visible, the subjects oriented towards their partner more both when it ate carrots and when it ate bananas than when it did not eat (Nothing vs. Carrot: *χ* ^2^ = 17.19, *df* = 1, *p* < 0.001; Nothing vs. Banana: *χ* ^2^ = 17.60, *df* = 1, *p* < 0.001; Figure 2a). These results confirm that the subject was aware that the partner had been given food.

Finally, we tested whether the subjects were distressed by seeing their partner eat. Visibility did not moderate the effect of food given to the partner on the rate of scratching (a behavioral indicator of anxiety‐like emotions; Maestripieri et al. 1992; Schino et al. 1996) shown by the subjects (interaction effect between Visibility and Nothing vs. Carrot: Coeff. = −0.04, *z* = −0.53, *N* = 278, *p* = 0.593; interaction effect between Visibility and Nothing vs. Banana: Coeff. = 0.01, *z* = 0.06, *N* = 278, *p* = 0.950; Supporting Information S1: Table S5 and Figure 2b). Considering only the test sessions in which the partner was visible, the subjects did not scratch more when their partner ate carrots or bananas than when they did not eat (Nothing vs. Carrot: *χ* ^2^ = 1.49, *df* = 1, *p* = 0.223; Nothing vs. Banana: *χ* ^2^ = 0.03, *df* = 1, *p* = 0.866; Figure 2b). Similarly, visibility did not moderate the effect of food given to the partner on cage shaking by the subjects (interaction effect between Visibility and Nothing vs. Carrot: Coeff. = 0.05, *z* = 0.03, *N* = 110, *p* = 0.978; interaction effect between Visibility and Nothing vs. Banana: Coeff. = 0.16, *z* = 0.44, *N* = 110, *p* = 0.657; Supporting Information S1: Table S6 and Figure 2c). Considering only the test sessions in which the partner was visible, the subjects did not show more cage shaking when the partner ate carrots or bananas than when it did not eat (Nothing vs. Carrot: *χ* ^2^ = 0.00, *df* = 1, *p* = 0.974; Nothing vs. Banana: *χ* ^2^ = 0.22, *df* = 1, *p* = 0.636; Figure 2c). Note that seven subjects were excluded from these latter analyses because they never showed cage shaking. Threats directed at the partner were extremely rare (only 5 events out of 278 test sessions), to the point that statistical analysis was impossible. Overall, these results suggest the subjects were not particularly stressed by seeing their partner eat.

### 3.2 Behavior During the Testing Phase

As already described in previous studies that used a similar experimental setting (de Waal 1997; Sabbatini et al. 2012; Schino, Ferrario, et al. 2021), during our tests food sharing was in most cases passive (i.e., the partner reached through the wire mesh and collected pieces of food from the floor of the subject's cage) and peaceful. Threats directed at the subject were rare (only 8 events out of 278 test sessions).

Forced claims were similarly rare and occurred only in a single dyad. They were not included in the count of food transfers in the following analyses.

**Figure 3:**
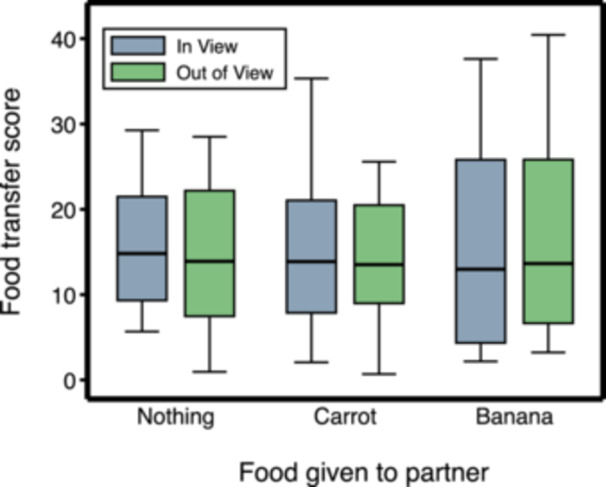
Food transfers from the subject to the partner during the Testing Phase in relation to food eaten by the partner during the Experimental Treatment Phase (box plots showing medians, interquartile ranges, and adjacent values). The food transfer score is calculated as the number of food transfers per 100 g of food available for sharing (see the text for details).

**Figure 4:**
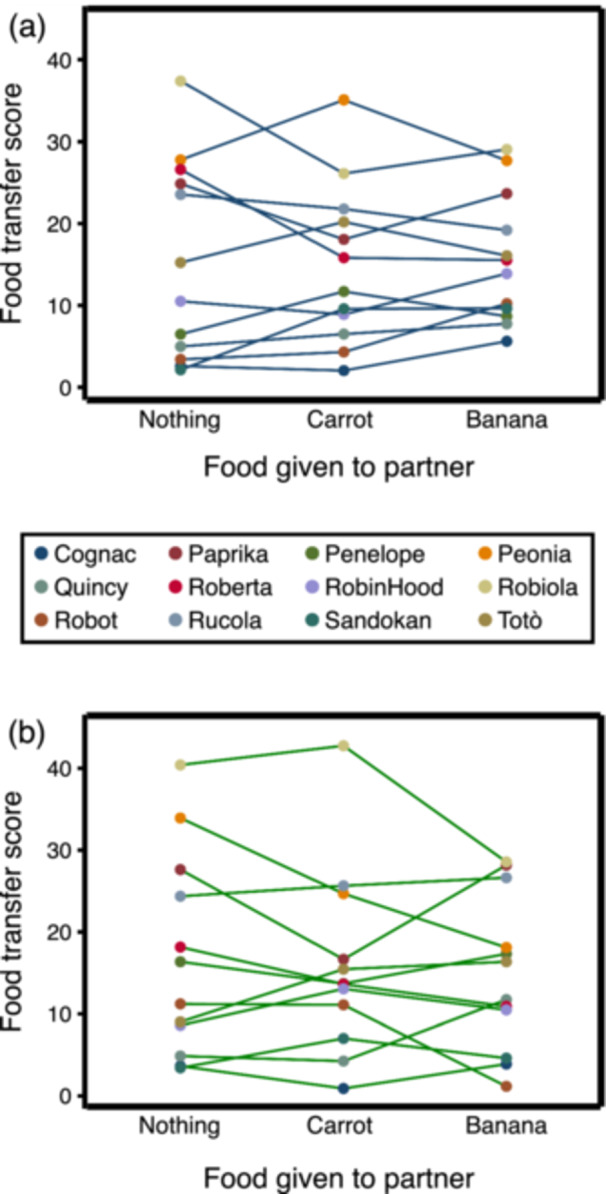
Individual data on food transfers from the subject to the partner during the Testing Phase in relation to food eaten by the partner during the Experimental Treatment Phase. (a) When the partner had eaten in view of the subject; (b) When the partner had eaten out of view of the subject.

We first tested whether, during the Testing Phase, the subjects altered their willingness to allow partners to obtain some of their food in relation to having seen them eat during the Experimental Treatment Phase. Visibility of the partner did not moderate the effect of food eaten by partners during the Experimental Treatment Phase on subsequent food transfers (interaction effect between Visibility and Nothing vs. Carrot: Coeff. = 0.06, *z* = 0.46, *N* = 278, *p* = 0.645; interaction effect between Visibility and Nothing vs. Banana: Coeff. = 0.15, *z* = 0.93, *N* = 278, *p* = 0.351; Supporting Information S1: Table S7 and Figure 3). Considering only the test sessions in which the partner was visible during the Experimental Treatment Phase, food transfers did not increase when the partner had received no food compared to when it had received carrot or banana (Nothing vs. Carrot: *χ* ^2^ = 0.11, *df* = 1, *p* = 0.735; Nothing vs. Banana: *χ* ^2^ = 0.62, *df* = 1, *p* = 0.432; Figures 3 and 4).

We reasoned that, even when the partner was visible during the Experimental Treatment Phase, subjects may have been differently aware of their partner's food consumption depending on the time actually spent looking at the partner. We therefore repeated the analysis above, controlling for the proportion of time the subject spent oriented to the partner during the Experimental Treatment Phase (this analysis obviously included only test sessions in which the partner was visible during the Experimental Treatment Phase). The results did not change (Nothing vs. Carrot: Coeff. = 0.02, *z* = 0.21, *N* = 139, *p* = 0.832; Nothing vs. Banana: Coeff. = −0.08, *z* = −0.95, *N* = 139, *p* = 0.343; Supporting Information S1: Table S8).

To make our test more similar to that conducted by Hattori et al. (2012), we repeated the analyses above, including only specific subsets of data. Following their experimental design, we included in the analysis only the first test session for each experimental condition, only female‐female dyads, only food transfers that occurred in view of the subject, or only the test sessions in which the partner had spent at least 70% (or 90%) of time in the experimental cages. Nevertheless, doing so did not change our results. None of the tests resulted in any significant difference in food transfers in relation to the subject having seen the partner eat during the Experimental Treatment Phase (Supporting Information S1: Tables S9–S13).

Finally, we tested whether subjects interpreted failed attempts to collect food as signals of need, and if they responded accordingly. Food transfers were not significantly related to failed attempts to collect food (Coeff. = −0.14, *z* = −0.60, *N* = 278, *p* = 0.546; Supporting Information S1: Table S14).

## 4 Discussion

In this study, we aimed at testing whether capuchin monkeys altered their willingness to share food with a group companion in relation to having seen it eat a more or a less preferred food. Our capuchin monkeys seemed aware that their partner was eating (thus demonstrating task understanding), and did not show any sign of distress (thus showing little emotional response to the unequal allocation of resources). When subsequently given a shareable food, capuchins allowed their partner to obtain some of their food irrespective of the partner's previous food consumption. The quality of the food consumed by the partner did not seem to affect the immediate emotional response of the subject or its subsequent food sharing, suggesting the emotional/motivational hypothesis of Hattori et al. (2012) did not apply to our sample of capuchin monkeys.

Clearly, any study testing cooperativeness in relation to the need for cooperation relies on the assumption that the subject is motivated to ameliorate the state of need of a partner. This assumption is certainly reasonable in some settings (e.g., food provisioning to a mate during the breeding season in monogamous birds), but should always be carefully evaluated. With regard to our study, we know that capuchin monkeys readily share food under the experimental conditions we used, and that this food sharing is not based on selfish motivations such as the expectation of reciprocation or the desire to improve one's own reputation (Schino, Boggiani, et al. 2021; Schino, Ferrario, et al. 2021). When given the choice, capuchin monkeys prefer to share food with partners with whom they have a better relationship (Sabbatini et al. 2012), possibly indicating that some concern for the benefit of their partner may be involved. It is also worth noting that in none of the test sessions did the subject consume all of the food (food remaining in the cup ranged from 21 to 146 g), suggesting that subjects were not reluctant to share their food irrespective of the partner's state of need because they always wanted all the food for themselves. Finally, our use of “compatible” pairs, while limiting the generalizability of our results, also reduced the risk that subjects were simply too hostile to their partner to care about their needs.

The results of our study confirm some aspects of food sharing in capuchin monkeys reported in Hattori et al. (2012) and in studies conducted in our and others' labs (e.g., de Waal 1997; Schino, Boggiani, et al. 2021; Schino, Ferrario, et al. 2021). Capuchin monkeys shared readily and peacefully food through a mesh, but when this was not possible, the monkey who did not eat did not show any sign of distress. However, differences in the units of measurement used in the different studies make it impossible to compare quantitatively the amount of food shared. Despite this general agreement, our results do not confirm those of Hattori et al. (2012) on the effect of having seen a partner eat on the subsequent willingness to share food. They reported reduced food sharing after the observation of the partner's food consumption, while we found no such effect.

It should be noted that several significant differences exist between the two studies. For example, in contrast to Hattori et al. (2012) (and for reasons explained in the Introduction), we chose to use different types of food in the experimental treatment and in the testing phases. Differently from Hattori et al. (2012), our subjects did not have access to food before the test. Our “Nothing” condition differed from the equivalent condition in Hattori et al. (2012) in that we did not give any food to the partner, while in their study, food was present but was unavailable to the partner because of a transparent partition (we reasoned that the more the different experimental conditions were clearly distinguishable, the easier the task would be). Also, our test sessions were overall shorter, and we gave partners and subjects different amounts of food. Finally, all of our female‐female dyads were related, which likely affected their tolerance. Some of these discrepancies may have significantly impacted our results, and our study should not be considered a full replication of that by Hattori et al. (2012).

At least two explanations are therefore possible for the differences between the results of the two studies. First, it is possible that the observed differences simply reflect the inevitable interindividual variability that exists in every biological population and/or the vagaries of statistical sampling. Second, it is possible that the differences in the details of the experimental procedures of the two studies affected their results. During data analysis, we tried to further approach what had been done by Hattori et al. (2012). For example, since they had conducted only one test session per subject and experimental condition, we included only the first test session per subject and experimental condition into these new analyses, and since all of their subjects were female, we included only female‐female dyads. The results based on these subsets of data confirmed those obtained on the whole data set. At the moment, it is impossible to say whether the remaining differences between the two studies played a role in determining their different results. Studies investigating the effects of knowledge about the partner state of hunger/satiation on food sharing in other taxa also provided inconsistent evidence (see Section 1) and point again to small and/or not very robust effects. Overall, our understanding of the ability of animals to comprehend the internal states of others is still fragmentary and inconclusive.

The available evidence derived from the present and previous studies seems to indicate that the effect of having seen another individual eat on the willingness to share part of one's own food is either small and variable (and thus difficult to detect) or sensitive to variations in the experimental setup (and thus not very robust). It is also interesting to compare the conflicting results about the understanding of a partner's state of hunger/satiety with two other sociocognitive characteristics of capuchin monkeys that have been repeatedly tested: their ability to reciprocate food sharing and their aversion to inequity. Regarding reciprocation, repeated experiments (although presenting inevitable methodological differences) have yielded consistent results confirming the ability of capuchin monkeys to reciprocate food sharing in an experimental setting similar to that used in this study (de Waal 2000; Sabbatini et al. 2012; Schino, Ferrario, et al. 2021). In contrast, studies investigating inequity aversion (i.e., the tendency to refuse a lower quality reward if a partner has received a better reward for the same task) have yielded variable results that seem at least in part be associated with apparently minor details of the experiment or of the housing conditions (see Ritov et al. 2024 for a meta‐analysis and Schweinfurth and Call 2021 for a review of the factors affecting the response to inequity). Even within a single, relatively well‐studied species, findings regarding some aspects of the social life (and the underlying cognitive characteristics) have been consistently reported, while others seem more difficult to replicate.

The variability in the available evidence about the ability of animals to understand (and act to ameliorate) the internal state of a partner warrants some general considerations about the importance of replication in animal behavior research. The current “replication crisis” (Pashler and Wagenmakers 2012; Open Science Collaboration 2015) is often responded to by invoking greater transparency and openness of scientific practices. While laudable, these initiatives seem to imply that if the results of a study cannot be replicated, then something must be “wrong” in the original study (McNutt 2014). This view ignores the variability inherent in any biological phenomenon and underestimates our ignorance about the factors that may modulate behavioral responses to any experimental treatment (see Amodio et al. 2021 for a similar argument). In contrast, we believe solid scientific knowledge can only be obtained on the basis of: (1) (multiple) replications of experimental and observational findings; (2) (systematic) variation of the experimental and observational conditions; (3) absence of selective reporting; (4) meta‐analyses based on multiple studies testing the same general hypothesis. This is an inevitably slow process, and our study (replicating, with variations, a previous experiment and reporting nonsignificant findings) is just a small step in this long exercise of knowledge accumulation.

## Author Contributions

**Gabriele Schino:** conceptualization, data curation, formal analysis, investigation, methodology, visualization, writing – original draft, writing – review and editing. **Guendalina Francesconi:** data curation, investigation, visualization, writing – review and editing. **Elsa Addessi:** conceptualization, investigation, methodology, writing – review and editing.

## Ethics Statement

Our research protocol was approved by the Italian Health Ministry (Central Direction for Veterinary Services, approval n. 335/2017‐PR), and we followed the ASAB/ABS Guidelines for the Use of Animals in Research and the American Society of Primatologists (ASP) Principles for the Ethical Treatment of Non‐Human Primates. Housing conditions and experimental procedures complied with the European law on the protection of animals used for scientific purposes (Directive 2010/63/EU).

## Conflicts of Interest

The authors declare no conflicts of interest.

## Supporting information

UndNeed AJP esm3.

UndNeed AJP StataCode3.

## Data Availability

The data and code that support the findings of this study are available in the supporting information of this article.
