# Supplementary material for: Does Knowledge of the Partner's Need Affect Food Sharing in Tufted Capuchin Monkeys?
Source: Am J Primatol. 2025 Oct 24;87(10):e70083. doi: 10.1002/ajp.70083 (PMC12551362; doi:10.1002/ajp.70083)
Supplement: Supplementary file 1 — UndNeed AJP esm3. [file AJP-87-e70083-s001.docx]

Does knowledge of the partner's need affect food sharing in tufted capuchin monkeys?

Gabriele Schino, Guendalina Francesconi and Elsa Addessi

SUPPLEMENTARY METHODS

| **Behavior** | **Definition** | **Test phase** | **Sampling method** |
| --- | --- | --- | --- |
| Threat to other | One monkey stares at the other with retracted and flattened ears, and raised forehead and eyebrows; generally, the tail is raised as well. | Both phases | Complete record |
| Cage shaking/banging | Shaking the cage bars or banging on the cage bars or the partition. | Both phases | Complete record |
| Scratching | A (usually) repeated movement of the hand or foot during which the fingertips are drawn across an individual's fur. | Both phases | Complete record |
| Subject orientation | The subject is oriented towards the partner or not | Experimental Treatment Phase | Instantaneous sampling (15 s) |
| Partner handling/eating food | The partner is handling or eating food or not | Experimental Treatment Phase | Instantaneous sampling (15 s) |
| Collect food in view | The partner collects pieces of food from the floor of the subject's cage through the mesh partition, in full view of the subject. | Testing Phase | Complete record |
| Collect food out of view | The partner collects pieces of food from the floor of the subject's cage through the mesh partition, out of view of the subject (i.e., while the subject is oriented elsewhere). | Testing Phase | Complete record |

*Table continues next page*

| Relaxed claim | The partner reaches through the mesh and removes some food directly from the subject's hands or mouth in a relaxed manner without aggression or use of force. | Testing Phase | Complete record |
| --- | --- | --- | --- |
| Forced claim | The partner takes food out of the subject's hands or mouth despite resistance. | Testing Phase | Complete record |
| Attempted collect food in view | The partner tries to collect pieces of food from the floor of the subject's cage through the mesh partition, in full view of the subject, but fails. | Testing Phase | Complete record |
| Attempted collect food out of view | The partner tries to collect pieces of food from the floor of the subject's cage through the mesh partition, out of view of the subject (i.e., while the subject is oriented elsewhere), but fails. | Testing Phase | Complete record |
| Attempted relaxed claim | The partner reaches through the mesh and tries to remove some food directly from the subject’s hands or mouth in a relaxed manner without aggression or use of force, but fails. | Testing Phase | Complete record |
| Attempted forced claim | The partner tries forcefully to take food out of the subject’s hands or mouth, but fails. | Testing Phase | Complete record |
| Food giving | The subject appears to deliberately drop, push, or throw food through the mesh partition in the partner's cage, or to give or push food with its hands or mouth to the partner in a way that seems directed at the other. | Testing Phase | Complete record |
| Accidental food giving | The subject drops food through the mesh partition, but as the result of messy food consumption or food dumping. | Testing Phase | Complete record |
| Partner out of experimental cages | The partner exits the experimental cages. | Both phases | Complete record |

Table S1. Behaviors recorded during the study.

SUPPLEMENTARY RESULTS

**Food Preferences**

Before running the experiment, we evaluated the preferences of capuchin monkeys for the different types of food to be used during the experiment. We wanted to be sure that our subjects had clear preferences among the different foods. Specifically, our experimental procedure required that apple (the food received by the subjects during the Testing Phase) was more and less preferred, respectively, than carrot and banana (the two different types of food received by the partner during the Experimental Treatment Phase). We presented each subject with two equally-sized pieces of food (either one of carrot and one of apple, or one of banana and one of apple). After the subject had taken one of the two pieces, we prevented it from taking the other. The procedure was repeated 40 times divided into two test sessions, and we recorded which food was chosen in each trial.

All subjects preferred banana over apple and apple over carrot in at least 80% of trials.

**Details of Statistical Analyses**

Tables S2 to S14 report the complete regression tables relative to all analyses included in the main text, plus the additional analyses conducted on subsets of data in order to approximate analyses conducted by Hattori et al. (2012). Tables include information on sample sizes and on all dependent and independent variables inserted into analyses. In the Tables below, by "Amount of food available for sharing" we mean the amount of food given to the subject (150 g) minus the amount of food remaining in the cup at the end of the test.

Behavior during the Experimental Treatment Phase

**Table S2.** Modulation by being visible to the subject of the effect of type of food received on amount of food consumed by the partner. Test sessions in which no food was given to the partner were excluded from this analysis.

**Data included into analysis**: All test sessions, excluding those in which no food was given to the partner

**Dependent variable**: Amount of food taken from the bowl

**Indep. variables Coefficient *z* *P***

Food Type (Carrot vs. Banana) 20.72 6.22 <0.001

Visibility -1.30 -0.62 0.536

Visibility × Carrot vs. Banana 1.11 0.51 0.607

Intercept 29.00 8.71 <0.001

Within-subject (fixed effect) linear regression with bootstrap standard errors (10000 replications); N=184 test sessions.

**Contrasts analysis**

(Visibility: In View; Food Type: Carrot) vs. (Visibility: In View; Food Type: Banana):

𝜒^2^=38.69, df=1, P<0.001

**Table S3.** Modulation by being visible to the subject of the effect of type of food received on time spent eating by the partner. Test sessions in which no food was given to the partner were excluded from this analysis.

**Data included into analysis**: All test sessions, excluding those in which no food was given to the partner

**Dependent variable**: Count of sampling points in which the partner ate or handled food

**Indep. variables Coefficient *z* *P***

Food Type (Carrot vs. Banana) -0.03 -0.20 0.838

Visibility 0.00 -0.04 0.967

Visibility × Carrot vs. Banana -0.01 -0.18 0.859

**Exposure variable**: Total number of sampling points

Within-subject (fixed effect) conditional Poisson regression with bootstrap standard errors (10000 replications); N=184 test sessions.

**Contrasts analysis**

(Visibility: In View; Food Type: Carrot) vs. (Visibility: In View; Food Type: Banana):

𝜒^2^=0.04, df=1, P=0.838

**Table S4.** Modulation by visibility of the effect of food received by the partner on time the subject spent oriented towards the partner.

**Data included into analysis**: All test sessions

**Dependent variable**: Count of sampling points in which the subject was oriented towards the partner

**Indep. variables Coefficient *z* *P***

Food Type (Nothing vs. Carrot) 0.16 4.15 <0.001

Food Type (Nothing vs. Banana) 0.26 4.19 <0.001

Visibility 0.01 0.10 0.918

Visibility × Nothing vs. Carrot -0.25 -2.02 0.044

Visibility × Nothing vs. Banana -0.07 -0.82 0.414

**Exposure variable**: Total number of sampling points

Within-subject (fixed effect) conditional Poisson regression with bootstrap standard errors (10000 replications); N=278 test sessions.

**Contrasts analysis**

(Visibility: In View; Food Type: Nothing) vs. (Visibility: In View; Food Type: Carrot):

𝜒^2^=17.19, df=1, P<0.001

(Visibility: In View; Food Type: Nothing) vs. (Visibility: In View; Food Type: Banana):

𝜒^2^=17.60, df=1, P<0.001

**Table S5.** Modulation by visibility of the effect of food received by the partner on scratching by the subject.

**Data included into analysis**: All test sessions

**Dependent variable**: Count of scratching by the subject

**Indep. variables Coefficient *z* *P***

Food Type (Nothing vs. Carrot) 0.07 1.22 0.223

Food Type (Nothing vs. Banana) 0.01 0.17 0.866

Visibility -0.06 -0.85 0.396

Visibility × Nothing vs. Carrot -0.04 -0.53 0.593

Visibility × Nothing vs. Banana 0.01 0.06 0.950

**Exposure variable**: Duration of the experimental treatment phase

Within-subject (fixed effect) conditional Poisson regression with bootstrap standard errors (10000 replications); N=278 test sessions.

**Contrasts analysis**

(Visibility: In View; Food Type: Nothing) vs. (Visibility: In View; Food Type: Carrot):

𝜒^2^=1.49, df=1, P=0.223

(Visibility: In View; Food Type: Nothing) vs. (Visibility: In View; Food Type: Banana):

𝜒^2^=0.03, df=1, P=0.866

**Table S6.** Modulation by visibility of the effect of food received by the partner on cage shaking by the subject.

**Data included into analysis**: All test sessions (but seven subjects had to be excluded because they never showed any cage shaking)

**Dependent variable**: Count of cage shaking by the subject

**Indep. variables Coefficient *z* *P***

Food Type (Nothing vs. Carrot) 0.06 0.03 0.974

Food Type (Nothing vs. Banana) 0.16 0.47 0.636

Visibility 0.10 0.74 0.457

Visibility × Nothing vs. Carrot 0.05 0.03 0.978

Visibility × Nothing vs. Banana 0.16 0.44 0.657

**Exposure variable**: Duration of the experimental treatment phase

Within-subject (fixed effect) conditional Poisson regression with bootstrap standard errors (10000 replications); N=110 test sessions.

**Contrasts analysis**

(Visibility: In View; Food Type: Nothing) vs. (Visibility: In View; Food Type: Carrot):

𝜒^2^=0.00, df=1, P=0.974

(Visibility: In View; Food Type: Nothing) vs. (Visibility: In View; Food Type: Banana):

𝜒^2^=0.22, df=1, P=0.636

Behavior during the Testing Phase

**Table S7.** Modulation by visibility of the effect of food received by the partner during the Experimental Treatment Phase on subsequent food transfers.

**Data included into analysis**: All test sessions

**Dependent variable**: Count of food transfers

**Indep. variables Coefficient *z* *P***

Food Type (Nothing vs. Carrot) 0.02 0.34 0.735

Food Type (Nothing vs. Banana) -0.07 -0.79 0.432

Visibility -0.03 -0.21 0.830

Visibility × Nothing vs. Carrot 0.06 0.46 0.645

Visibility × Nothing vs. Banana 0.15 0.93 0.351

**Exposure variable**: Amount of food available for sharing

Within-subject (fixed effect) conditional Poisson regression with bootstrap standard errors (10000 replications); N=278 test sessions.

**Contrasts analysis**

(Visibility: In View; Food Type: Nothing) vs. (Visibility: In View; Food Type: Carrot):

𝜒^2^=0.11, df=1, P=0.735

(Visibility: In View; Food Type: Nothing) vs. (Visibility: In View; Food Type: Banana):

𝜒^2^=0.62, df=1, P=0.432

**Table S8.** Effect of food received by the partner during the Experimental Treatment Phase on subsequent food transfers, controlling for time spent by the subject oriented to the partner and including only test sessions in which the partner had been visible during the Experimental Treatment Phase.

**Data included into analysis**: Only test sessions in which the partner had been visible during the Experimental Treatment Phase

**Dependent variable**: Count of food transfers

**Indep. variables Coefficient *z* *P***

Food Type (Nothing vs. Carrot) 0.02 0.21 0.832

Food Type (Nothing vs. Banana) -0.08 -0.95 0.343

Time oriented to partner 0.15 0.26 0.791

**Exposure variable**: Amount of food available for sharing

Within-subject (fixed effect) conditional Poisson regression with bootstrap standard errors (10000 replications); N=139 test sessions.

**Table S9.** Modulation by visibility of the effect of food received by the partner during the Experimental Treatment Phase on subsequent food transfers. The analysis includes, for each subject, only the first test session for each experimental condition.

**Data included into analysis**: For each subject, only the first test session for each experimental condition

**Dependent variable**: Count of food transfers

**Indep. variables Coefficient *z* *P***

Food Type (Nothing vs. Carrot) 0.08 0.46 0.646

Food Type (Nothing vs. Banana) 0.08 0.24 0.807

Visibility -0.33 -1.18 0.238

Visibility × Nothing vs. Carrot 0.22 0.76 0.446

Visibility × Nothing vs. Banana 0.32 0.62 0.538

**Exposure variable**: Amount of food available for sharing

Within-subject (fixed effect) conditional Poisson regression with bootstrap standard errors (10000 replications); N=72 test sessions.

**Contrasts analysis**

(Visibility: In View; Food Type: Nothing) vs. (Visibility: In View; Food Type: Carrot):

𝜒^2^=0.21, df=1, P=0.646

(Visibility: In View; Food Type: Nothing) vs. (Visibility: In View; Food Type: Banana):

𝜒^2^=0.06, df=1, P=0.807

**Table S10.** Modulation by visibility of the effect of food received by the partner during the Experimental Treatment Phase on subsequent food transfers. The analysis includes only female-female dyads.

**Data included into analysis**: Female-female dyads only

**Dependent variable**: Count of food transfers

**Indep. variables Coefficient *z* *P***

Food Type (Nothing vs. Carrot) 0.07 1.09 0.278

Food Type (Nothing vs. Banana) -0.02 -0.27 0.788

Visibility 0.09 0.56 0.574

Visibility × Nothing vs. Carrot 0.04 0.25 0.801

Visibility × Nothing vs. Banana 0.15 0.78 0.434

**Exposure variable**: Amount of food available for sharing

Within-subject (fixed effect) conditional Poisson regression with bootstrap standard errors (10000 replications); N=120 test sessions.

**Contrasts analysis**

(Visibility: In View; Food Type: Nothing) vs. (Visibility: In View; Food Type: Carrot):

𝜒^2^=1.18, df=1, P=0.278

(Visibility: In View; Food Type: Nothing) vs. (Visibility: In View; Food Type: Banana):

𝜒^2^=0.07, df=1, P=0.788

**Table S11.** Modulation by visibility of the effect of food received by the partner during the Experimental Treatment Phase on subsequent food transfers. The analysis includes only food transfers that occurred in view of the subject (i.e., it excluded all instances of "Collect out of view"; see Table S1).

**Data included into analysis**: All test sessions

**Dependent variable**: Count of food transfers that occurred in view of the subject

**Indep. variables Coefficient *z* *P***

Food Type (Nothing vs. Carrot) -0.01 -0.08 0.934

Food Type (Nothing vs. Banana) -0.18 -1.92 0.055

Visibility -0.05 -0.35 0.729

Visibility × Nothing vs. Carrot 0.12 0.91 0.362

Visibility × Nothing vs. Banana 0.20 1.21 0.225

**Exposure variable**: Amount of food available for sharing

Within-subject (fixed effect) conditional Poisson regression with bootstrap standard errors (10000 replications); N=278 test sessions.

**Contrasts analysis**

(Visibility: In View; Food Type: Nothing) vs. (Visibility: In View; Food Type: Carrot):

𝜒^2^=0.01, df=1, P=0.934

(Visibility: In View; Food Type: Nothing) vs. (Visibility: In View; Food Type: Banana):

𝜒^2^=3.68, df=1, P=0.055

**Table S12.** Modulation by visibility of the effect of food received by the partner during the Experimental Treatment Phase on subsequent food transfers. The analysis includes only test sessions in which the partner spent at least 70% of time in the experimental cages.

**Data included into analysis**: Only test sessions in which the partner spent at least 70% of time in the experimental cages

**Dependent variable**: Count of food transfers

**Indep. variables Coefficient *z* *P***

Food Type (Nothing vs. Carrot) 0.05 0.73 0.465

Food Type (Nothing vs. Banana) 0.01 0.16 0.873

Visibility 0.05 0.38 0.702

Visibility × Nothing vs. Carrot -0.02 -0.15 0.879

Visibility × Nothing vs. Banana 0.12 0.63 0.530

**Exposure variable**: Amount of food available for sharing

Within-subject (fixed effect) conditional Poisson regression with bootstrap standard errors (10000 replications); N=164 test sessions.

**Contrasts analysis**

(Visibility: In View; Food Type: Nothing) vs. (Visibility: In View; Food Type: Carrot):

𝜒^2^=0.53, df=1, P=0.465

(Visibility: In View; Food Type: Nothing) vs. (Visibility: In View; Food Type: Banana):

𝜒^2^=0.03, df=1, P=0.873

**Table S13.** Modulation by visibility of the effect of food received by the partner during the Experimental Treatment Phase on subsequent food transfers. The analysis includes only test sessions in which the partner spent at least 90% of time in the experimental cages.

**Data included into analysis**: Only test sessions in which the partner spent at least 90% of time in the experimental cages

**Dependent variable**: Count of food transfers

**Indep. variables Coefficient *z* *P***

Food Type (Nothing vs. Carrot) 0.13 1.46 0.145

Food Type (Nothing vs. Banana) 0.16 1.02 0.307

Visibility 0.03 0.22 0.826

Visibility × Nothing vs. Carrot -0.01 -0.05 0.959

Visibility × Nothing vs. Banana 0.17 0.66 0.507

**Exposure variable**: Amount of food available for sharing

Within-subject (fixed effect) conditional Poisson regression with bootstrap standard errors (10000 replications); N=103 test sessions.

**Contrasts analysis**

(Visibility: In View; Food Type: Nothing) vs. (Visibility: In View; Food Type: Carrot):

𝜒^2^=2.13, df=1, P=0.145

(Visibility: In View; Food Type: Nothing) vs. (Visibility: In View; Food Type: Banana):

𝜒^2^=1.04, df=1, P=0.307

**Table S14.** Relation between failed attempts by the partner to collect food in view of the subject and food transfers, controlling for failed attempts to collect food out of view of the subject and for the different experimental conditions.

**Data included into analysis**: All test sessions

**Dependent variable**: Count of food transfers

**Indep. variables Coefficient *z* *P***

Failed attempts in view of subject -0.01 -0.60 0.546

Failed attempts out of view of subject 0.02 0.47 0.636

Food Type (Nothing vs. Carrot) 0.03 0.62 0.533

Food Type (Nothing vs. Banana) -0.02 0.72 0.496

Visibility 0.05 0.72 0.469

**Exposure variable**: Amount of food available for sharing

Within-subject (fixed effect) conditional Poisson regression with bootstrap standard errors (10000 replications); N=278 test sessions.
