# Supplementary material for: Does Knowledge of the Partner's Need Affect Food Sharing in Tufted Capuchin Monkeys?
Source: Am J Primatol. 2025 Oct 24;87(10):e70083. doi: 10.1002/ajp.70083 (PMC12551362; doi:10.1002/ajp.70083)
Supplement: Supplementary file 2 — UndNeed AJP StataCode3. [file AJP-87-e70083-s002.docx]

Does knowledge of the partner's need affect food sharing in tufted capuchin monkeys?

Gabriele Schino, Guendalina Francesconi and Elsa Addessi

**Stata code used in the analyses**

Before running the analyses, all string variables were transformed into numeric variables using the encode command.

Stata code used to obtain the results shown in Table S2

. xtset Subject_n

. xtreg FoodTaken_Pre ExpCond_Food3_n##ExpCond_View_n if ExpCond_Food3_n > 1, fe vce(bootstrap, reps(10000) seed(1) dots(100))

. contrast r.ExpCond_Food3_n@ExpCond_View_n

Stata code used to obtain the results shown in Table S3

. xtset Subject_n

. xtpoisson Ea_Pre_P_Y ExpCond_Food3_n##ExpCond_View_n if ExpCond_Food3_n > 1, fe exposure(SamplPoint_Pre) vce(bootstrap, reps(10000) seed(1) dots(100))

. contrast r.ExpCond_Food3_n@ExpCond_View_n

Stata code used to obtain the results shown in Table S4

. xtset Subject_n

. xtpoisson Or_Pre_S_Y i.ExpCond_Food3_n##ExpCond_View_n, fe exposure(SamplPoint_Pre) vce(bootstrap, reps(10000) seed(1) dots(100))

. contrast r.ExpCond_Food3_n@ExpCond_View_n

Stata code used to obtain the results shown in Table S5

. xtset Subject_n

. xtpoisson St_Pre_S ExpCond_Food3_n##ExpCond_View_n, fe exposure(Pretest_durat) vce(bootstrap, reps(10000) seed(1) dots(100))

. contrast r.ExpCond_Food3_n@ExpCond_View_n

Stata code used to obtain the results shown in Table S6

. xtset Subject_n

. xtpoisson CS_Pre_S ExpCond_Food3_n##ExpCond_View_n, fe exposure(Pretest_durat) vce(bootstrap, reps(10000) seed(1) dots(100))

. contrast r.ExpCond_Food3_n@ExpCond_View_n

Stata code used to obtain the results shown in Table S7

. xtset Subject_n

. xtpoisson FoodTransf ExpCond_Food3_n##ExpCond_View_n, fe exposure(FoodTaken100_Test) vce(bootstrap, reps(10000) seed(1) dots(100))

. contrast r.ExpCond_Food3_n@ExpCond_View_n

Stata code used to obtain the results shown in Table S8

. xtset Subject_n

. xtpoisson FoodTransf i.ExpCond_Food3_n Or_Pre_S_prop if ExpCond_View_n==1, fe exposure(FoodTaken100_Test) vce(bootstrap, reps(10000) seed(1) dots(100))

Stata code used to obtain the results shown in Table S9

. xtset Subject_n

. xtpoisson FoodTransf ExpCond_Food3_n##ExpCond_View_n if Test_N_1_4==1, fe exposure(FoodTaken100_Test) vce(bootstrap, reps(10000) seed(1) dots(100))

. contrast r.ExpCond_Food3_n@ExpCond_View_n

Stata code used to obtain the results shown in Table S10

. xtset Subject_n

. xtpoisson FoodTransf ExpCond_Food3_n##ExpCond_View_n if FF_only==1, fe exposure(FoodTaken100_Test) vce(bootstrap, reps(10000) seed(1) dots(100))

. contrast r.ExpCond_Food3_n@ExpCond_View_n

Stata code used to obtain the results shown in Table S11

. xtset Subject_n

. xtpoisson FT_InView ExpCond_Food3_n##ExpCond_View_n, fe exposure(FoodTaken100_Test) vce(bootstrap, reps(10000) seed(1) dots(100))

. contrast r.ExpCond_Food3_n@ExpCond_View_n

Stata code used to obtain the results shown in Table S12

. xtset Subject_n

. xtpoisson FoodTransf ExpCond_Food3_n##ExpCond_View_n if OoV_Test_P_prop<=0.3, fe exposure(FoodTaken100_Test) vce(bootstrap, reps(10000) seed(1) dots(100))

. contrast r.ExpCond_Food3_n@ExpCond_View_n

Stata code used to obtain the results shown in Table S13

. xtset Subject_n

. xtpoisson FoodTransf i.ExpCond_Food3_n##ExpCond_View_n if OoV_Test_P_prop<=0.1, fe exposure(FoodTaken100_Test) vce(bootstrap, reps(10000) seed(1) dots(100))

. contrast r.ExpCond_Food3_n@ExpCond_View_n

Stata code used to obtain the results shown in Table S14

. xtset Subject_n

. xtpoisson FoodTransf AttemptFT_InView ACo_Test_P i.ExpCond_Food3_n ExpCond_View_n, fe exposure(FoodTaken100_Test) vce(bootstrap, reps(10000) seed(1) dots(100))
